# First insights into the impacts of benthic cyanobacterial mats on fish herbivory functions on a nearshore coral reef

**DOI:** 10.1038/s41598-021-84016-z

**Published:** 2021-03-30

**Authors:** Amanda K. Ford, Petra M. Visser, Maria J. van Herk, Evelien Jongepier, Victor Bonito

**Affiliations:** 1 School of Agriculture, Geography, Environment, Ocean and Natural Sciences (SAGEONS), University of the South Pacific, Suva, Fiji; 2 Stockholm Resilience Centre, Stockholm University, Stockholm, Sweden; 3 Department of Freshwater and Marine Ecology, Institute for Biodiversity and Ecosystem Dynamics, University of Amsterdam, Amsterdam, The Netherlands; 4 Bioinformatics, Institute for Biodiversity and Ecosystem Dynamics, University of Amsterdam, Amsterdam, The Netherlands; 5 Reef Explorer Fiji, Korolevu, Fiji

**Keywords:** Ecology, Ecological genetics, Microbial ecology, Tropical ecology, Marine biology

## Abstract

Benthic cyanobacterial mats (BCMs) are becoming increasingly common on coral reefs. In Fiji, blooms generally occur in nearshore areas during warm months but some are starting to prevail through cold months. Many fundamental knowledge gaps about BCM proliferation remain, including their composition and how they influence reef processes. This study examined a seasonal BCM bloom occurring in a 17-year-old no-take inshore reef area in Fiji. Surveys quantified the coverage of various BCM-types and estimated the biomass of key herbivorous fish functional groups. Using remote video observations, we compared fish herbivory (bite rates) on substrate covered primarily by BCMs (> 50%) to substrate lacking BCMs (< 10%) and looked for indications of fish (opportunistically) consuming BCMs. Samples of different BCM-types were analysed by microscopy and next-generation amplicon sequencing (16S rRNA). In total, BCMs covered 51 ± 4% (mean ± s.e.m) of the benthos. Herbivorous fish biomass was relatively high (212 ± 36 kg/ha) with good representation across functional groups. Bite rates were significantly reduced on BCM-dominated substratum, and no fish were unambiguously observed consuming BCMs. Seven different BCM-types were identified, with most containing a complex consortium of cyanobacteria. These results provide insight into BCM composition and impacts on inshore Pacific reefs.

## Introduction

Though scarcely mentioned in the literature a decade ago, benthic cyanobacterial mats (BCMs) are receiving increasing attention from researchers and managers as being a nuisance on tropical coral reefs worldwide^1–4^. Whereas BCMs were known to bloom seasonally at some locations, the prevalence and duration of them are increasing at an alarming rate (see review^1^). Though in part this observation can be linked to observers being more aware of them, a unique 40-year dataset from the Caribbean showed unequivocally that they have become more dominant in recent years alongside declines in hard corals and other key benthic groups^5^. The factors directly responsible for these changes remain uncertain but decreasing water quality and increasing water temperatures are likely primarily responsible^2,6–8^. Indeed, cyanobacteria are projected to become more problematic in a variety of aquatic systems in the coming years with increasing climate change-related factors and deteriorating local conditions that favour their growth^9–11^.

Similarly to their better-studied freshwater counterparts^12^, proliferation of BCMs in coral reef ecosystems are associated with a wide range of problems due to their fast-growing opportunistic nature and their intrinsic toxic properties^1^. BCMs are notoriously competitive in interactions with corals, and often result in damage or mortality of coral tissue (e.g. Brown et al.^13^). They can also overgrow other benthic species due to their fast growth rates and have been reported to smother organisms or cause stress due to the rich variety of secondary metabolites they produce^14–16^. BCMs can inhibit coral larval settlement and survival^17,18^, though effects appear to be species-specific, with high variation among different taxa (e.g. of corals, cyanobacteria)^19^. They are often reported to bloom during warmer summer months which commonly correspond to spawning times, when larvae are seeking suitable substrate to settle on. Given that successful coral recruitment and survivorship is critical for reef recovery dynamics^20^, BCM proliferation can be expected to seriously compromise the resilience of coral reefs to acute and chronic disturbances. They also release high amounts of bioavailable nitrogen^21,22^ as many taxa fix nitrogen, and are one of the biggest benthic contributors of dissolved organic carbon^23^ (DOC; i.e. sugars), thus further stimulating fast-growing primary producers and/or microorganisms and fuelling negative feedback loops^24^.

Despite the recent surge in reports of BCM proliferation, there are still many gaps in basic knowledge, including their composition, the specific factors that facilitate their growth, and their impact on ecological processes and functions. Furthermore, in spite of the vast literature on reef fish diets and grazing preferences, little is known about BCM consumption by reef fish. In contrast, macroalgae (which have more commonly been implicated as the ‘villain’ on degraded reefs) have been comprehensively studied in terms of their effect on coral recruitment and survivorship^25,26^, outcomes of their interactions with hard corals^27,28^, and their palatability to a broad diversity of reef fish species (e.g. Rasher et al.^29^). Accordingly, it is widely accepted that management of specific functional groups or species of fish that consume macroalgae can assist in their reduction (i.e. top-down control). The limited research into BCM consumption tends to indicate that top-down control of mats by fish is rather minimal (e.g. Capper et al.^30^), implying that species-specific management would be futile in reducing them. Nonetheless, two recent observational studies have yielded some interesting insights into mat consumption. One study on Australia’s Great Barrier Reef observed *Bolbometapon muricatum* feeding on mats^31^, and another on the Caribbean island of Bonaire observed some species of angelfish (*Holacanthus tricolor, Pomacanthus paru*), parrotfish (*Scarus coeruleus, Scarus iseri*), and surgeonfish (*Acanthurus bahianus, Acanthurus coeruleus*), appearing to take bites from mats^32^; though notably, ascertaining whether fish consume the cyanobacteria themselves, or are rather targeting trapped detritus, sediment, or other associated fauna is impossible through observations alone.

While identifying reef species that may (opportunistically) consume mats is clearly important for management planning, of equal importance is understanding how BCM proliferation impacts reef fish processes at the reef scale. Herbivory is known to be critical for ecological resilience but is impeded at most tropical coral reefs due to human-mediated impacts such as overfishing and sedimentation^33–35^. Reefs in the Pacific Island region have much higher fish and benthic species richness than other areas such as the Red Sea and the Caribbean, facilitating higher functional redundancy (i.e. many species perform the same function^36^) and greater response diversity (i.e. a large diversity of responses to ecosystem changes among species within a functional group^37^), making this region particularly interesting to measure ecological responses to ecosystem changes such as BCM proliferation.

Here, we investigate a natural BCM bloom at an inshore no-take marine protected area on Fiji’s Coral Coast. First, we quantified the benthic and fish communities at the study site during the seasonal BCM bloom and classified the different BCM-types observed based on their morphology and colouration. Second, we investigated how BCM proliferation impacted herbivory functions using remote video observations over natural grazable substrates dominated *versus* devoid of BCMs. Third, we assessed any indications of fish species (opportunistically) consuming BCMs from videos overlooking BCM-dominated substrate. Based on prior observations, we hypothesised that the presence of BCMs would impede herbivory and that BCMs themselves would not be consumed at a level that would indicate top-down control. Finally, we collected samples of dominant BCM-types and identified their cyanobacterial composition using a combination of microscopy and amplicon sequencing. To the best of our knowledge, this is both the first study into BCMs in Fiji and the first to document how BCMs impact fish herbivory in a natural, diverse in situ environment.

## Results

### Survey data

**Figure 1:**
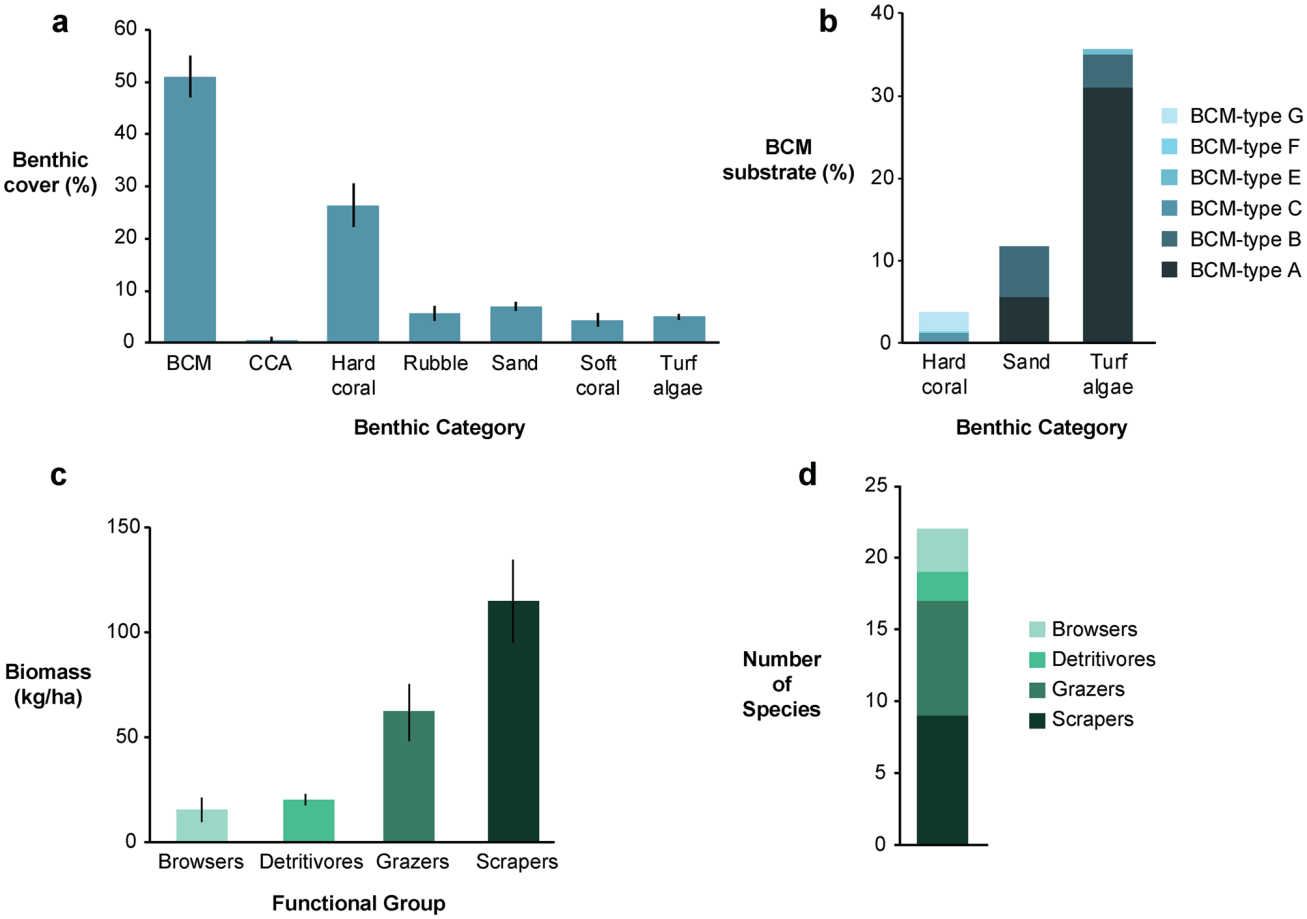
Survey data from the study site within the Namada no-take area: (**a**) benthic cover determined by benthic point-intercept transects (mean ± s.e.m.), (**b**) relative proportion of substrate types over which BCMs were growing, (**c**) herbivorous fish biomass by functional group according to stationary point counts (mean ± s.e.m.), and (**d**) number of species observed over all stationary point counts by functional group. BCM = benthic cyanobacterial mat, CCA = crustose coralline algae.

Benthic transects revealed that BCMs dominated the study area in February/March 2019 (51 ± 4%; mean ± s.e.m.) (Fig. 1a). Most BCMs were growing over turf algae (70 ± 10% of total BCM cover)—either turf-covered pavement (51 ± 9%) or rubble (19 ± 5%)—followed by sand (23 ± 5%), and live hard coral (7 ± 2%) (Fig. 1b). After BCMs, hard coral was the next most common benthic group (29 ± 4%) (Fig. 1a). BCMs covered 77% of the grazable substrate at the study site (when considering grazable substrate as [turf-covered] rubble and pavement—total 47% of the study site), and in total 14 ± 4% of the hard coral at the site was overgrown by BCMs. No macroalgae were recorded on benthic transects.

**Figure 2:**
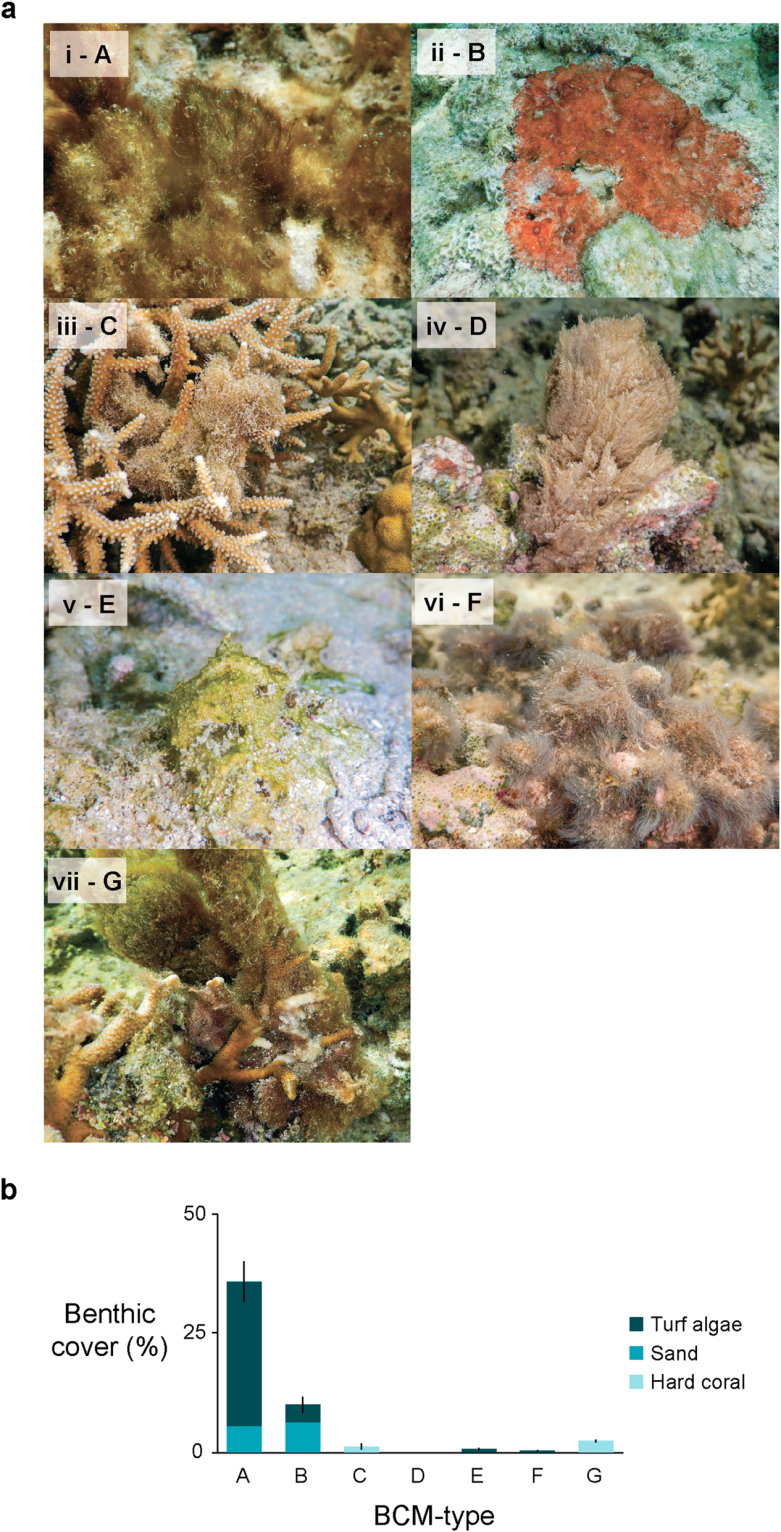
Photographs (**a**) of the different BCM-types (i–vii, corresponding to BCM-types ‘A–G’ throughout) observed at the study site in February/March 2019, with (**b**) their benthic cover (mean ± s.e.m. as quantified on benthic transects) over different substrates (hard coral, sand, turf algae). BCM-type labels on the x-axis (A, B, C, D, E, F, G) in (**b**) correspond to labels in part (**a**).

Seven distinct BCM morphologies, described in Table 1 and hereon referred to as BCM-types A–G, were observed at the study site (Fig. 2a i–vii corresponding to BCM-types A–G; Table 1). While most BCM-types had a strictly mat morphology (including the most common types A and B) three BCM-types (C, D, G) were observed growing more as ‘clumps’ than mats (see descriptions in Table 1), but because of their ability to sprawl along the benthos they are hereon also referred to as ‘BCM-types’. All but one BCM-type (BCM-type D) were measured in benthic transects (Fig. 2b). The different BCM-types showed preferences for different substrates, with BCM-types A and B consistently found growing over turf algae and sand, BCM-types E and F only on turf algae, and BCM-types C and G only on hard coral (Fig. 2b).

**Table 1:** BCM-types (A–G; refer to Fig. 2 for photographs), descriptions of their morphology, and lists of taxa identified by microscopic analysis.

BCM-type	Coverage in surveys (%)	Description	Taxa identified by microscopy
A	37	Forms thick, extensive mats over non-coral substrate. Generally, brown/red in colour	*Lyngbya* s.l., *Anabaena* cf*., Calothrix, Phormidium* cf*., Spirulina, Tychonema, Chroococcus* cf.
B	10	Grows like a thin mat over sand and hard substrate. Has a slightly ‘rubbery’ feel and can peel off the substrate in fragile rubbery pieces. Has a distinctive red/maroon colour. Often has air bubbles on its surface and observed peeling off the substrate on which it grows	*Lyngbya* s.l., *Anabaena* cf*., Leptolyngbya, Spirulina*
C	1	Forms thick, ‘fluffy’ mats that grow primarily on scleractinian corals. Generally, reddish/brown to golden in colour. Mats are thick and can sprawl off the corals they are growing on	*Lyngbya* s.l.
D	0	Forms thick, puff balls that become mats when abundant. Grows primarily on scleractinian corals. Consistently light brown in colour. Occasionally woven by shrimp into tubes within coral skeletons	*Lyngbya* s.l*., Oscillatoria, Phormidioideae*
E	1	A thin, fuzzy mat that grows over sand and occasionally hard substrate. Consistently green in colour	*Anabaena* cf*., Lyngbya* s.l*., Oscillatoria*
F	0.2	Forms a thin, fluffy mat consisting of fine strands that grow over hard substrate. Consistently grey in colour	*Lyngbya* s.l*., Calothrix, Heteroleibleinia, Oscillatoria, Phormidioideae*
G	2	Forms thick and heavy balls that float off of hard-bottom substrate and are attached by narrow strands at the bottom. Generally brown to reddish in colour	*Lyngbya* s.l., *Heteroleibleinia, Calothrix*

Stationary point counts of the herbivorous fish assemblage at the site recorded an overall herbivorous fish biomass of 212 ± 36 kg ha^−1^. The lowest biomass values were recorded for browsers (16 ± 6 kg ha^−1^), followed by detritivores (20 ± 3 kg ha^−1^), grazers (62 ± 14 kg ha^−1^), and then scrapers (115 ± 20 kg ha^−1^) (Fig. 1c). Three browser species were detected, two detritivores, eight grazers, and eight scrapers (plus some juveniles that were only classified to genus level due to difficulties in identifying to species) (Fig. 1d). No excavators were detected in stationary point counts.

### Video observations

**Figure 3:**
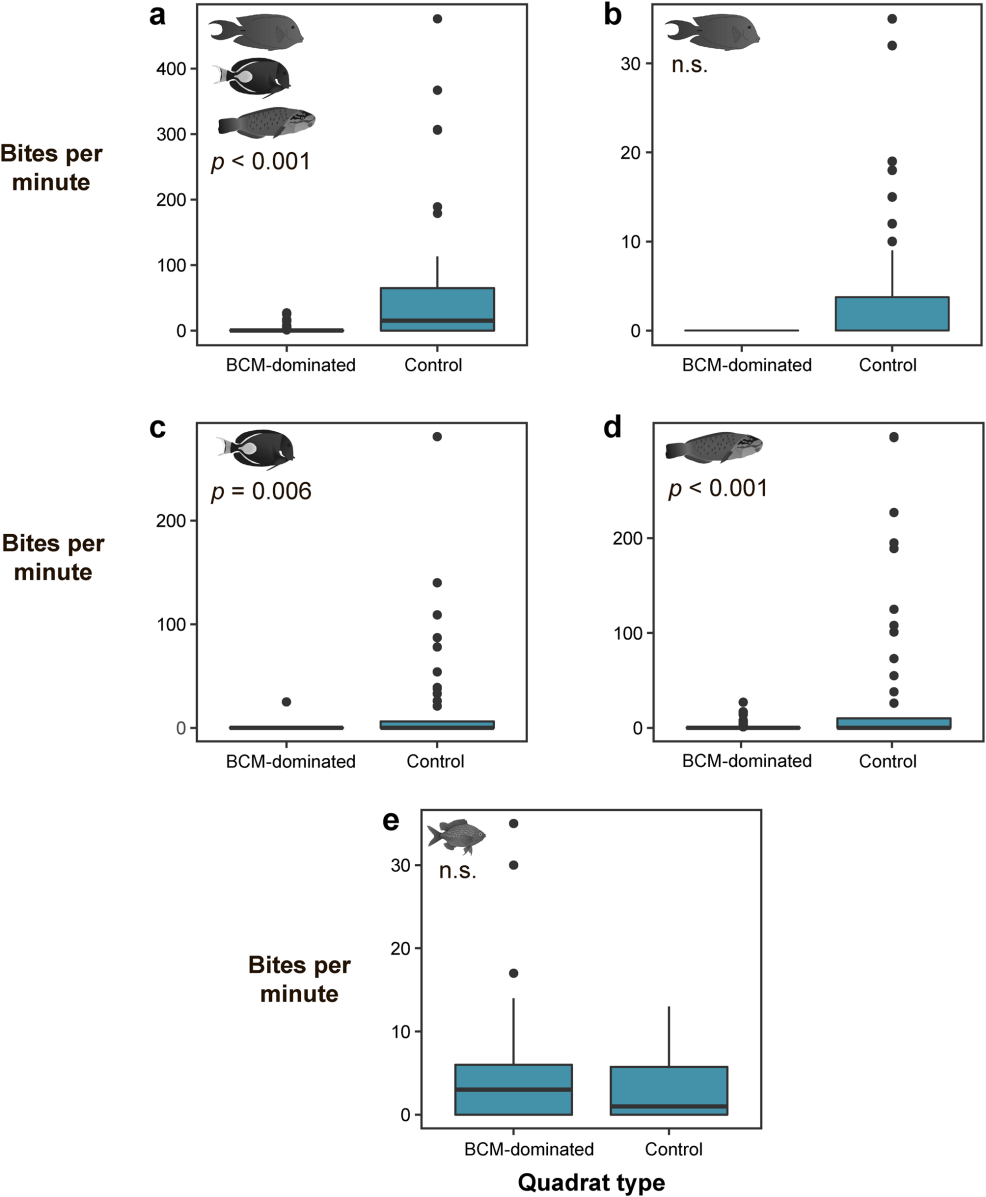
Boxplots representing bite rates measured in quadrats dominated by benthic cyanobacterial mats (‘BCM-dominated’) or devoid of mats (‘Control’). Plots represent bites on the substrate observed for: (**a**) all herbivorous functional groups combined (i.e. sum of b–d), (**b**) detritivores, (**c**) grazers, (**d**) scrapers, and (**e**) damselfishes. Significance level as determined by zero-inflated generalised linear mixed effect models is shown for each graph; n.s. = not significant. Note the different scales on the y-axis.

Overall herbivorous fish bite rates were significantly lower on substrates dominated by BCMs (1 ± 0.3 bites m^−2^ substrate min^−1^) compared with control substrates without BCMs (59 ± 15 bites m^−2^ substrate min^−1^) (GLMM, *p* < 0.001; Fig. 3a). While there was not a significant difference in bite rates between the two surfaces for detritivores, notably they were never observed to feed in quadrats dominated by BCMs (compared to 4 ± 1 bites m^−2^ substrate min^−1^ on control surfaces; Fig. 3b). Grazers were observed to take significantly more bites on control substrates without mats (19 ± 7 bites m^−2^ substrate min^−1^) compared to substrates where BCMs dominated (0.1 ± 0.1 bites m^−2^ substrate min^−1^) (GLMM, *p* = 0.006; Fig. 3c), as did scrapers (36 ± 11 bites m^−2^ substrate min^−1^ and 1 ± 0.3 bites m^−2^ substrate min^−1^; respectively) (GLMM, *p* < 0.001; Fig. 3d). Finally, damselfish bite rates did not differ between BCM-dominated substrate and control substrate without BCMs, but unlike detritivores, damselfish were observed biting on both substrate treatments (Fig. 3e).

From 180 min of video observations of BCM-dominated substrates, with the exception of some small site-attached damselfishes, no fish were unambiguously observed to feed directly on mats. In instances where an individual could conceivably have taken one bite on a mat, they never took a second bite.

### BCM-types and composition

#### Microscopy

**Figure 4:**
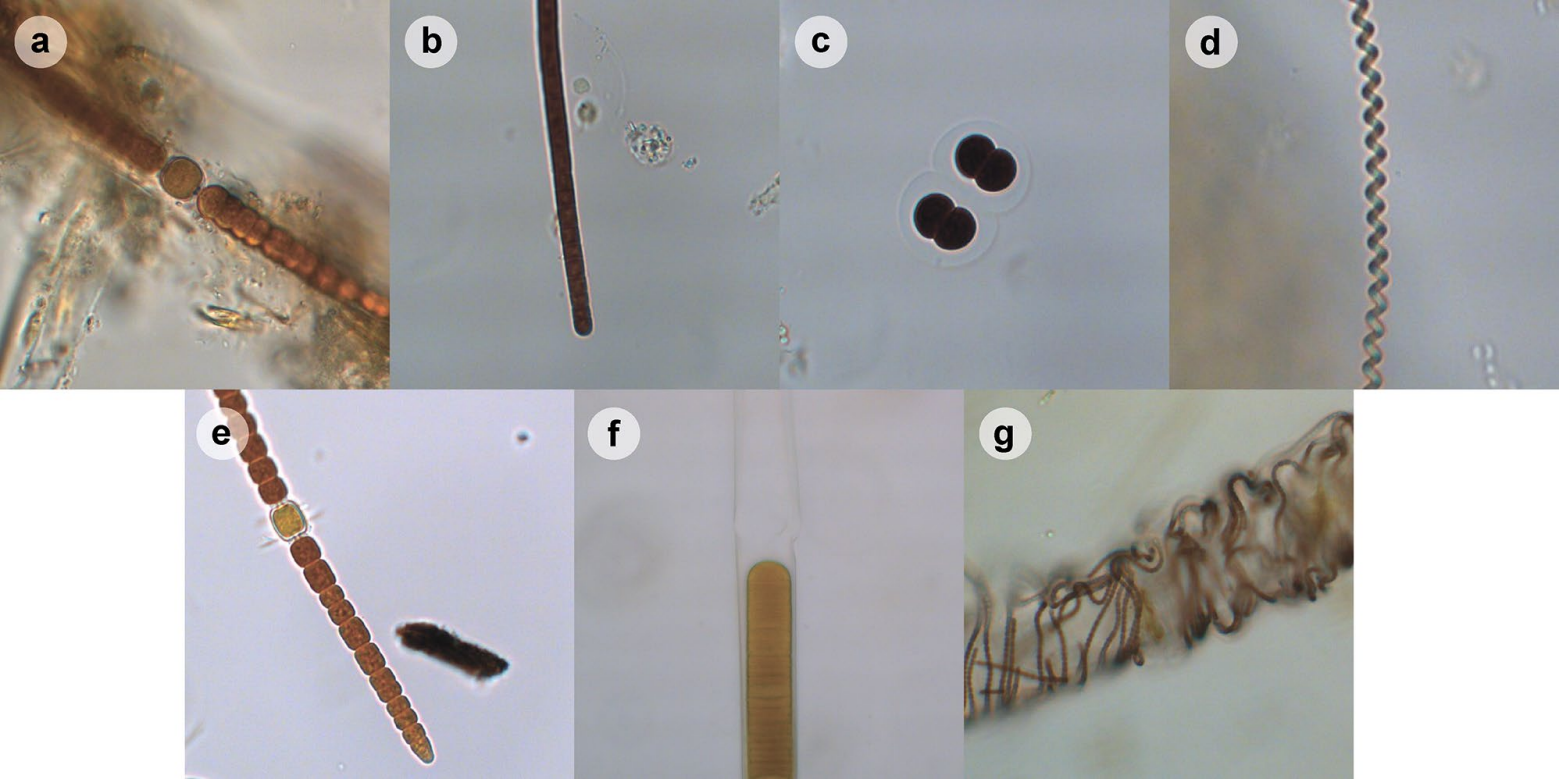
Microscopy images of the most common individual cyanobacterial taxa identified from across the seven BCM-types: (**a**) *Calothrix*, (**b**) *Oscillatoriales*, (**c**) *Chroococcus* cf., (**d**) *Spirulina*, (**e**) *Anabaena* cf., (**f**) *Lyngbya* s.l., (**g**) *Heteroleibleinia*. All pictures were taken from Lugol’s preserved samples. Photographs a–e are at 630× magnification, and f–g are at 400× magnification.

All BCM-types, with the exception of type E, were dominated by filaments that identify as *Lyngbya, Okeania,* or *Moorea* (see Table 1 for taxa identified by microscopy; see Fig. 4 for representative images of common cyanobacterial taxa). These three genera cannot be distinguished from each other through bright field microscopy^38^ and are therefore referred to as *Lyngbya* s.l. ([sensu lato], in a broad sense, as opposed to *Lyngbya* s.s. [sensu stricto], in a narrow sense). BCM-type E was dominated by filaments resembling *Anabaena*, which were also abundant in BCM-type A. Microscopy identified different combinations of cyanobacterial taxa in other BCM-types. The taxa *Calothrix, Heteroleibleinia, Oscillatoria*, *Phormidioideae*, and *Spirulina* were detected in more than one BCM-type, whereas *Phormidium* cf., ([confer], resembling closely but identity not confirmed) *Tychonema*, and *Chroococcus* cf*.* (all detected in BCM-type A only) as well as *Leptolyngbya* (BCM-type B) were observed in single BCM-types. According to microscopy, BCM-type A was the most diverse, and BCM-type C was the least diverse with only *Lyngbya* s.l. detected.

#### Next-generation amplicon sequencing

**Figure 5:**
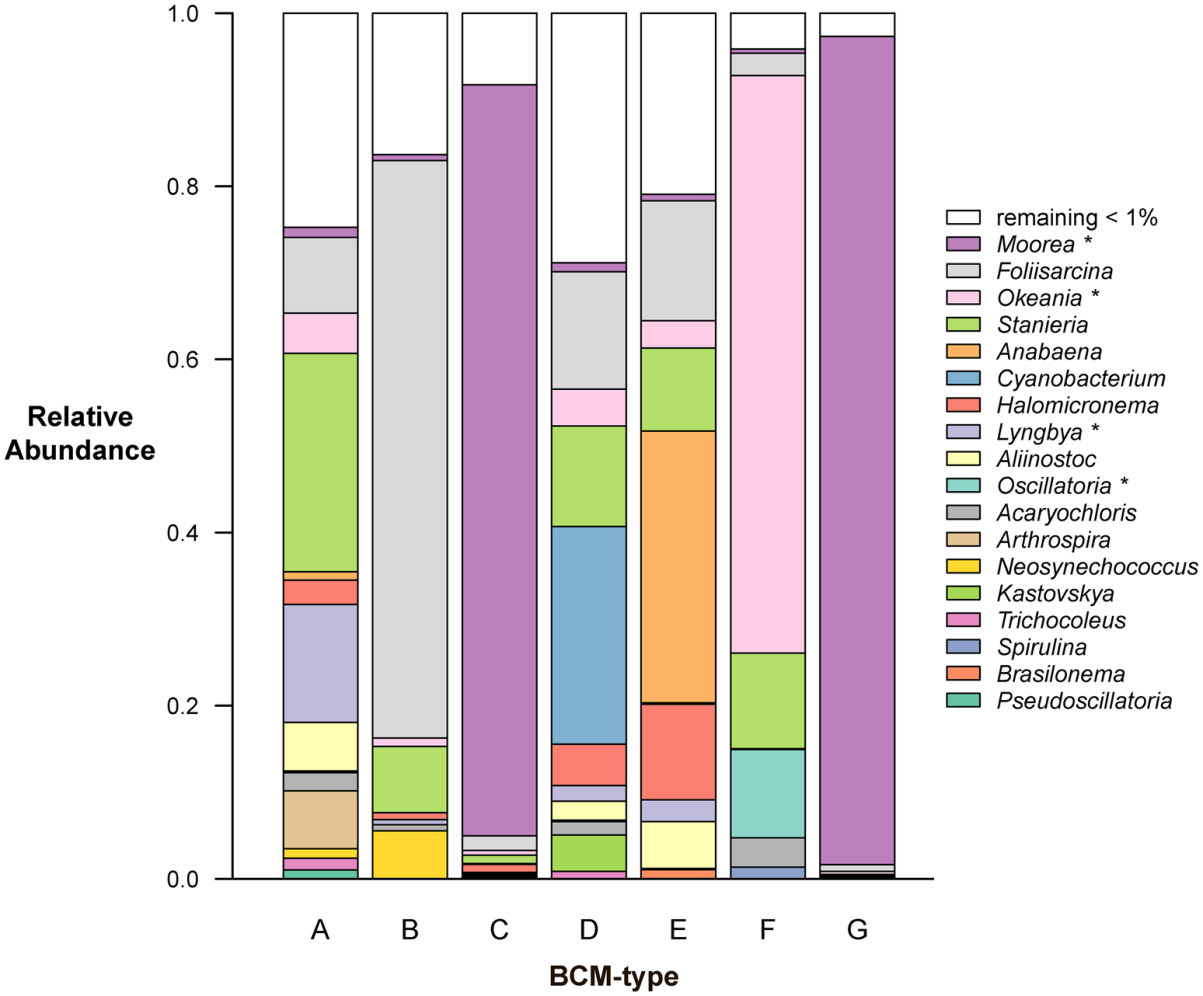
Relative abundance of cyanobacterial genera as quantified by amplicon sequencing and identified using BLAST analysis. The genera are named after the best-blast hit. Genus names with * had a similarity of > 95%, while all other genera had a lower similarity and are most likely a different, unknown genus. BCM-type labels on the x-axis (A, B, C, D, E, F, G—correspond to Fig. 2 and Table 1). N.B. *Pseudoscillatoria* is named in NCBI as *Roseofilum*^65^.

Sequencing data revealed a large variety of cyanobacterial genera in most of the BCM-types (Fig. 5), with some taxa abundant in some mats and not in others, some mats showing high diversity, and generally little overlap between mats (see heatmap in Fig. S1). According to the BLAST analysis, the cyanobacteria belonged to 18 genera (% reads in any sample > 1%). The genera *Moorea*, *Okeania*, and *Oscillatoria* had very high percentages of sequence similarity (> 99%, > 97%, and 100%, respectively) according to the BLAST analysis (Table 2). All others had percentages of similarity close to or below 95%. Only BCM-types C and G showed > 90% dominance (i.e. 94 and 97%, respectively; referring to relative proportion of cyanobacterial reads) of one cyanobacterial genus, namely *Moorea* (Fig. 5; Fig. S1). BCM-type B was > 70% dominated by a genus which had the best-blast hit with *Foliisarcina—*this genus was also present in all other BCM-types but in a lower abundance. BCM-type F was ~ 65% dominated by *Okeania*. BCM-types D (one growing exclusively over hard coral) and E were comprised of a variety of genera with *Cyanobacterium* and *Anabaena* being of greatest abundance respectively (best-blast hits with an identity similarity of about 93%, Table 2).

**Table 2:** Best-blast hits of ASVs (with family and order) that occurred > 1% in at least one of the samples with percentage of query coverage and percentage identity (ID).

ASV ID	Best-Blast Hit	Family	Order	Query Coverage	% ID
FIJI 026; 031; 034	*Acaryochloris marina* MBIC11017	*Acaryochloridaceae*	Synechococcales	99–100%	90.2–91.4
FIJI 010; 047	*Aliinostoc morphoplasticum* strain NOS	*Nostocaceae*	Nostocales	100%	92.8–93.1
FIJI 005	*Anabaena cylindrica* PCC 7122	*Nostocaceae*	Nostocales	100%	93.1
FIJI 016	*Arthrospira platensis* strain PCC 7345	*Microcoleaceae*	Oscillatoriales	100%	92.8
FIJI 051	*Brasilonema octagenarum* strain UFV-E1	*Scytonemataceae*	Nostocales	100%	92.8
FIJI 006	*Cyanobacterium aponinum* strain PCC 10,605	*Cyanobacteriaceae*	Chroococcales	100%	93.3
FIJI 004; 007; 014; 020; 022; 036; 038; 046	*Foliisarcina bertiogensis* strain CENA333	*Xenococcaceae*	Pleurocapsales	99%	89.4–92.3
FIJI 012;019; 021; 049	*Halomicronema excentricum* strain TFEP1	*Prochlorotrichaceae*	Synechococcales	99–100%	91.2–94.1
FIJI 023	*Kastovskya adunca* strain ATA6-11-RM4	*Coleofasciculaceae*	Oscillatoriales	100%	94.9
FIJI 009	*Lyngbya aestuarii* PCC 7419	*Oscillatoriaceae*	Oscillatoriales	100%	95.5
FIJI 001; 002; 037; 040	*Moorea producens* strain 3L	*Oscillatoriaceae*	Oscillatoriales	100%	99.2–99.7
FIJI 017; 050	*Neosynechococcus sphagnicola* strain sy1	*Leptolyngbyaceae*	Synechococcales	100%	91.5–91.8
FIJI 003; 033; 039; 041; 042; 044; 045	*Okeania hirsuta* strain PAB-10-Feb-10–1	*Oscillatoriaceae*	Oscillatoriales	100%	97.9–99.7
FIJI 011	*Oscillatoria nigro-viridis* strain PCC 7112	*Oscillatoriaceae*	Oscillatoriaceae	100%	100
FIJI 052	*Pseudoscillatoria coralii* strain BgP10_4S (is *Roseofilum* sp.)	*Coleofasciculaceae*	Oscillatoriales	100%	94.7
FIJI 043	*Spirulina major* strain PCC 6313	*Spirulinaceae*	Spirulinales	99%	93.3
FIJI 008; 013; 015; 018; 024; 025; 027; 028; 029; 030; 035; 048	*Stanieria cyanosphaera* PCC 7437	*Dermocarpellaceae*	Pleurocapsales	99–100%	89.6–93.6
FIJI 032	*Trichocoleus desertorum* strain ATA4-8-CV2	*Trichocoleusaceae*	Synechococcales	100%	89.4

E-value < 1 E-131.The ASV ID is the code given to the different ASVs which can also be found in the phylogenetic tree (Fig. S2).

## Discussion

This study measured the abundance of BCMs during a natural seasonal bloom on a Fijian inshore reef system, quantified how the BCMs influenced fish herbivory, and examined the cyanobacterial composition of the different BCMs present on the reef using a combination of microscopic and genomic tools. We found BCMs were the most abundant group, covering over half of the benthos during the sampled bloom, though throughout most of the year their cover is negligible (V. Bonito, personal observation). Seven different BCM-types were observed and described, each of which was found to have a unique and rich diversity of taxa within them. Video observations indicated that herbivorous fish grazing and scraping functions are significantly reduced where BCMs have overgrown grazable substrate and yielded no evidence of fish feeding directly on BCMs despite the diverse assemblage of fish present on the study reef. We hereby discuss the findings of this novel study of BCMs on Fijian reefs and in situ quantification of BCM effects on herbivory with a diverse, healthy fish community.

Benthic surveys revealed that despite moderate coral cover and no macroalgae, BCMs were the dominant group within the benthic community at the time of the surveys, growing primarily over turf-dominated substrate. Importantly, over three-quarters of the grazable substrate (i.e. turf-covered rubble and pavement) of the reef was overgrown by BCMs. Aside from the BCMs, benthic composition at Namada was similar to that found at other nearby inshore reefs that have been protected from fishing pressure—well-grazed with minimal macroalgal cover, moderate hard coral cover, and turf algae as the dominant substrate cover^39,40^. Overall herbivorous fish biomass was ten-fold higher than a threshold identified below which shifts to algal-dominated reefs are more likely (i.e. 20 kg ha^−1^)^41^. With the exception of excavators, there was good representation of species across herbivorous functional groups.

Video observations revealed that BCM-dominance impedes fish herbivory on the substrate. To our knowledge this is the first study that documents the impact of BCMs on herbivory functions in situ on natural substrates. Previous studies have assessed the palatability of BCMs in laboratories and/or have focused on multi-choice feeding assays or examining the effect of their extracted secondary metabolites^30,42,43^. Remote video observations were incorporated into this study for two reasons; (i) to see what impact BCM dominance has on detritivore, grazing, and scraping fish functions (i.e. those that target detritus, turf algae, and microphytobenthos) and (ii) to get an indication if any fish species were (opportunistically) consuming BCMs. Remote video observations offer a brilliant tool to assess the functioning of the fish community while avoiding the interference of any in-water divers, and recent studies have used them to quantify herbivory functions across different environments^44,45^. Importantly, our findings do not prove that no organisms feed on BCMs—this would need a different study design that also measures invertebrate grazers—but they do suggest that opportunistic consumption by herbivorous reef fishes is minimal.

The impacts of BCMs on herbivory function will be dependent on the duration and extent of BCM blooms and the size of the reef. Our results do however suggest that as BCMs proliferate on reefs and expand their range, key ecological functions performed by herbivores could become impaired. Without a sufficient food source accessible in areas where BCM blooms persist, our results suggest that herbivorous fish would concentrate feeding on remaining grazable substrate, leading to higher competition for available resources, and potentially fish may ultimately be driven to leave and search for reef areas with higher food availability. Fish communities could also suffer directly from cyanobacterial blooms—a major die-off of juvenile rabbitfish *Siganus argenteus* and *Siganus spinus* attributed to starvation occurred as BCMs became dominant on coral reefs around Guam^46^. Reductions in diversity and abundance of fishes are known to be linked to coral mortality as a result of losses of topographic complexity and food availability among other factors, though herbivores have often been the most resilient to this due to constant or increasing availability of algae associated with coral decline^47–49^. If BCMs and not algae proliferate alongside coral loss, then impacts on herbivore communities may well be more conspicuous.

Reductions in herbivory functions, even for relatively short periods of time, could result in turf algal communities developing into longer uncropped filamentous algae, similar to those observed in territorial damselfish territories^50^. While short well-cropped turfs can facilitate coral settlement and can thus be considered conducive to coral recovery^51^, long turfs significantly impede coral settlement^52^, and are more competitive in interactions with hard corals^40^. Furthermore, long turf assemblages are more likely to trap sediments and create negative feedback loops in herbivory^34^. Not only are herbivory functions critical on reef ecosystems, but herbivores also constitute an important part of human diets in regions such as that of the Pacific islands, and thus factors that affect herbivore populations are important to consider for the social consequences.

Interestingly, when investigating the impact of bleaching-induced cyanobacteria proliferation on small coral-dwelling fish, researchers in Australia recently concluded there was no relationship between cyanobacteria and fish abundance at a local (1 m^2^) scale^53^. Accordingly, we found no difference in the presence and activity of damselfish between BCM-dominated substrates and controls, indicating that at least short-term dominance of mats may not negatively impact small site-attached reef fishes. Some of these damselfish may even have been taking bites from mats, though gut analyses^54^ would be required to determine whether they were consuming the cyanobacteria themselves rather than cleaning/removing detritus and/or sediment from their territory which we anticipate is more likely. Otherwise, no fish were observed to feed on BCMs during the video footage, suggesting that top-down control was limited at the study site. This is somewhat surprising given the high diversity of species detected in the study area, and the anticipated high response diversity to novel ecological states. As such we could have expected some species to opportunistically make use of the new dominant resource. These results also contrast to those of Cissell et al.^32^ who identified several species to be consuming mats in the Caribbean. Reasons for these differences could include the presence of different herbivorous fish species, because of difficulties in visually determining what fish are feeding on, or differences in mat composition and chemistry.

We identified seven distinct BCM-types at the study site and performed both microscopic and molecular analyses to gain first insights into the diversity they harbour. Both approaches have both their strengths and weaknesses, thus an integrated approach is recommended^55–57^. Traditional microscopic analyses have the advantage of requiring less sophisticated equipment but offer limited resolution because morphology can change with environment and distinction between species or genera is not always possible using morphological features. With molecular analyses, we are able to detect the presence of small cyanobacteria and distinguish between cryptotaxa, and thus generally identify more taxa than morphological analysis^58,59^. Examples of cryptotaxa include *Lyngbya* s.s*.*, *Moorea,* and *Okeania—*which are morphologically undistinguishable—as well as the genera that make up *Leptolyngbya* and *Synechococcus*^55^. On the other hand, morphotaxa have genetic uniformity and can only be distinguished by microscopy^57^.

We used amplicon sequence variants (ASVs) rather than operational taxonomic units in this study as Knight et al.^60^ recommend for microbiome analysis. Based on BLAST analysis (against the NCBI database) we could affiliate the ASVs to genera. The 16S rRNA dissimilarity-based identification of ASVs revealed that only four genera (*Moorea*, *Okeania*, *Lyngbya,* and *Oscillatoria*) met the criteria of a 95% cut-off of cyanobacterial genera delimitation that have previously been established (i.e. the best-blast hit with a similarity lower than 95% is probably not the genus that the ASV should be assigned to). Several ASVs annotated as *Moorea producens*, *Oscillatoria nigro-viridis,* and *Okeania hirsuta* met the 99% 16S similarity cut-off value to delimit species^38,61^. These findings are supported by the close proximity of these reference species to the associated ASVs in the phylogenetic tree (Fig. S2). However, we should be cautious with affiliating the ASVs to species level since the length of the marker gene that was sequenced was rather short (average 375 bp). All three are benthic marine species that have been found in tropical areas, e.g. *O. nigro-viridis* in Papua New Guinea^62^, *M. producens* also from Papua New Guinea but additionally found in the Caribbean^63^, and *O. hirsuta* in Okinawa^64^.

As expected, our results highlight common discrepancies between microscopy and molecular analyses reported in many studies^55,56,58,65–67^. The microscopic analyses indicated that all samples except BCM-type E were dominated by *Lyngbya* s.l., and molecular analyses identified this further for three mats: the cryptotaxon *Okeania* dominated BCM-type F whilst the cryptotaxon *Moorea* dominated BCM-types C and G. Though the low number of taxa in BCM-types C and G observed in the sequence analysis was in agreement with the microscopy, the sequence analysis found the other BCMs to have a much higher diversity of cyanobacterial genera. Overall, ten genera were identified by microscopy compared to 18 by genomic analysis. Coral reef BCMs composed of diverse cyanobacteria taxa have also been reported in other recent studies that integrated molecular analyses based on 16S sequencing^22,68^. Furthermore, whilst the genera *Oscillatoria*, *Spirulina*, *Anabaena* cf., and *Lyngbya* s.l. (i.e. *Moorea*, *Okeania,* and *Lyngbya* s.s.) that were identified by microscopy were also detected by BLAST analysis, this was not the case for *Calothrix*, *Phormidium*, *Tychonema*, *Leptolyngbya*, and *Heteroleibleinia*, either because of the absence of these species in the reference databases or because of inaccuracies distinguishing them morphologically.

Of the 18 ASVs detected in the sequence analysis, 14 of them showed a low similarity (< 95%) with affiliated genera indicating they could likely be new genera. For example, in BCM-types A, B, D, and E the contribution of a genus that was affiliated to *Foliisarcina* was quite high, but the affiliation to this genus had such a low similarity (~ 90%) that this ASV may be a new genus. The taxa identified as *Anabaena* cf. using both morphological and molecular approaches was previously unknown from marine ecosystems and could potentially be a new genus (Kaštovsky pers. comm. 2019). The clustering of the ASVs in the phylogenetic tree (Fig. S2) provides more information of other close lineages for the ASVs found in this study. Of course, the establishment of new genera requires further characterisation than minimal 16S dissimilarity criteria, but it suggests that tropical BCMs harbour a great deal of undiscovered diversity. As stated by Duperron et al.^38^, the 1700 described cyanobacterial species are only a subset of the actual diversity. Taxonomic studies of cyanobacteria have largely focused on freshwater pelagic cyanobacteria—which exhibit similar high diversity with many overlapping taxa to those identified here^12^—while tropical regions and marine benthic mats have been far less studied. New species and genera can be discovered in these habitats, which is of interest not only for the taxonomic but also chemical diversity^38^. Notably, it is also important to acknowledge that databases are incomplete and that low similarity may thus not necessarily reflect a novel genus and may be a result of the genus not being known to the reference database. Isolating and sequencing of cyanobacteria from mats would broaden our understanding of their composition, while the use of longer reads in sequencing would likely increase the resolution of the molecular methods^38^.

It is very likely that the mats we sampled produce a high diversity of metabolites. Genera such as *Lyngbya, Moorea,* and *Okeania* are known for being chemically-rich in toxic compounds^63,69^. However, the chemical diversity that exists in other taxa that have not been taxonomically described yet remains largely unknown. Freshwater BCMs are better-studied thus provide some insights into the wide diversity and impacts of associated toxins^12^, and emphasise the need to expand research on marine BCMs. It’s likely that differences in the metabolites produced by different BCMs, along with variation in tolerance to the metabolites by different fish species, can in part explain discrepancies in observations of grazing on BCMs between this study and others (e.g. Cissell et al.^32^).

This study provided a preliminary description of the cyanobacteria composition of BCMs blooming at our study site in 2019; the first study of this nature on BCMs in Fiji. As the results reveal most BCMs consist of a diversity of taxa and likely identified several new genera of cyanobacteria, it would seem worthwhile to further extend this work with sampling at more sites, habitats, and depths to assess the taxonomic and chemical diversity of BCMs in relation to environmental drivers. More extensive morphological and molecular analyses would expand our knowledge of BCM composition. Furthermore, for proper characterisation of new genera and species, isolation of strains would be essential. A polyphasic approach^65^, incorporating morphological, molecular, and ecological data, is needed to describe cyanobacteria diversity and would facilitate more robust comparisons across studies.

Despite the relative isolation of Pacific island reefs, they are by no means immune to the problems facing other tropical locations^70^. Coral cover is declining at many islands, with increasingly more reefs shifting into alternative regimes dominated by non-reef building organisms^71^, and BCMs are increasingly prevalent throughout the region as a result of iron input from shipwrecks, untreated sewage, and logging, among other factors^4,6,8,72^. For the last two years, mats were observed for the first time to endure throughout the colder months (May–August) at the study area. If BCMs become more of a permanent fixture at reefs, then our results suggest that herbivorous fish grazing will be concentrated in areas devoid of BCMs, potentially leading to increased competition for resources. We suggest that proliferation of BCMs on reefs may yield similar outcomes to increasing abundances of territorial damselfish due to reductions in herbivory functions and the creation of unfavourable biotic conditions for coral recruits. The potential for rollover effects on livelihoods and food security remains unknown. Finally, the results of this study further emphasise the need for reef surveys to differentiate between turf algae-covered substrate and BCM-covered substrate due to their vastly different ecological implications.

## Methodology

### Surveys

**Figure 6:**
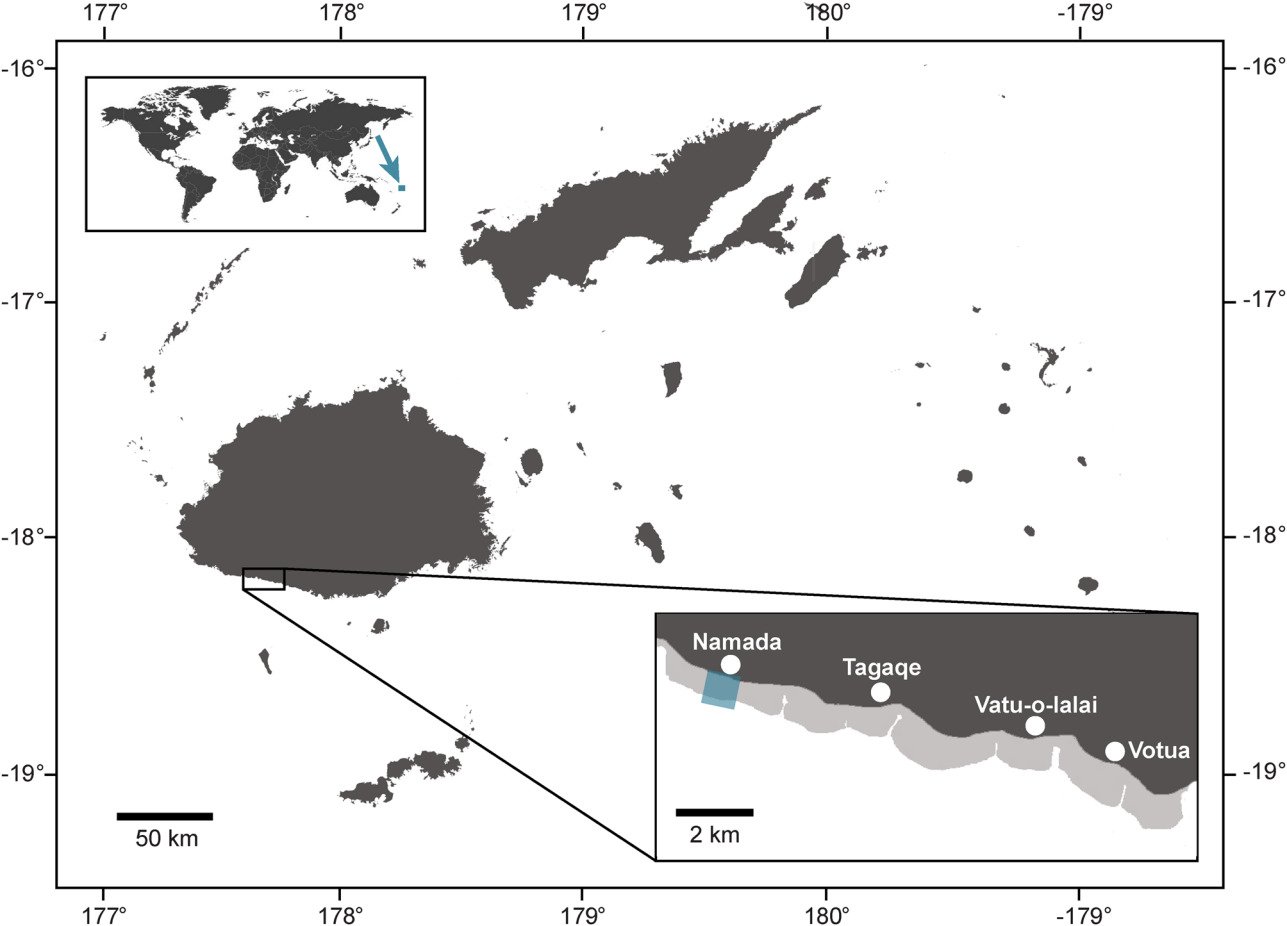
Map of Fiji, with study site (Namada no-take area) marked by blue rectangle. This figure was created using QGIS 3.4.9 (http://www.qgis.org).

Fieldwork took place in February and March 2019 within a small (< 0.7 km^2^), 17-year-old no-take marine protected area at Namada Village within the Korolevu-i-wai fishing ground along the Coral Coast of Fiji’s main island, Viti Levu (Fig. 6). The benthic community composition, including the cover of different BCM-types and what substrate BCMs were growing over, was quantified by a single observer using point-intercept transects (50-m in length; 100 points; n = 5). Surveys took place in the back of a reef flat moat at 1–3 m water depth. BCM-types were distinguished from each other by their distinct morphologies, textures, colourations, and overall appearances (Table 1) that held consistent across the study area.

Fish surveys were conducted by a single observer using the stationary point count method^73^. All fish observed in/passing through a 7.5 m radius cylinder around the observer were recorded over 5 min (n = 16 separate point counts conducted within the study area). Data were collected to species level and each fish’s total length was estimated to the nearest 2 cm. Biomass for each fish recorded in the survey was calculated using length–weight measurements and length conversion (when necessary) from Fishbase^74^ and presented as mean ± s.e.m. kg ha^−1^. Species were allocated to herbivorous functional groups (browsers, detritivores, excavators, grazers, scrapers) according to classifications in Heenan et al.^75^ (Table S1).

### Remote video observations and analysis

#### Video observations

To quantify how BCM dominance impacted the functioning of the herbivorous fish community, we compared bite (grazing) rates between substrate dominated by BCMs and substrate essentially devoid of BCMs in the same reef area where the benthic and fish surveys were conducted. GoPro cameras (GoPro Inc., USA) were fixed to dive weights and placed overlooking (i) substrate that was dominated by BCMs (> 50% cover) and (ii) ‘normal’ substrate that was not dominated by BCMs (< 10% cover) as a control. A 1 m^2^ quadrat frame was briefly placed in front of the GoPro camera and photographed from above to provide a birds-eye still image of the plot where herbivory data would be recorded. The quadrat frame was then removed, and the GoPro camera filmed continuously for the following *ca.* 60 min. Filming was conducted over four sequential days and started between 08:45 and 09:30. Each day, five GoPro cameras recorded grazing footage (n = 3 on BCM-dominated substrate; n = 2 on ‘normal’ turf-dominated [control] substrate) on different spots within the study area. After removing defective footage (i.e. foggy, bad quality, internal storage issues), eleven videos remained (n = 6 and n = 5 for BCM-dominated and control substrates, respectively).

Footage was visually analysed post hoc by a single observer. The quadrat area was first delineated in order to standardise bite rates within a known area (1 m^2^). For all videos, the first 20 min of footage was skipped to avoid any diver-related interference (with one exception where only 15 min was skipped to facilitate enough viewing prior to the video ending prematurely). For control videos, footage was analysed for the following 10 min (i.e. from 00:20:00 to 00:29:59), and for videos overlooking BCM-dominated substrate, footage was analysed for the following 30 min (i.e. from 00:20:00 to 00:49:59) (note: BCM-dominated videos were analysed for longer due to the additional objective to explore whether any fish species consumed mats, and due to quality limitations [such as increasing fogginess] reducing the reliability of later observations in some control videos—data were binned into 60-s intervals to avoid issues with unbalanced design; see “Statistical analysis” section). During this time, each individual fish taking a bite from the substrate within the delineated area was recorded (to the lowest resolution possible), and the number of consecutive bites taken was counted. A bite sequence ended upon the fish exiting the video frame.

For indications of fish feeding on BCMs, we evaluated 180 min (30 min clips; n = 6) of footage overlooking BCM-dominated substrate to record any fish seeming to take repetitive bites (≥ 2) of the BCMs. Though this approach does not confirm consumption, a fish taking more than one bite on BCM-dominated substrate could indicate that the species may be worth further investigating (e.g. through gut analyses) to explore its potential role in top-down control of mats.

#### Statistical analysis

For analysis, bites were categorised into those taken by the following herbivorous fish functional groups observed in the videos: detritivores (e.g. *Ctenachaetus* spp.), grazers (e.g. *Acanthuridae* spp.), and scrapers (e.g. *Scaridae* spp.). We also included the bites of Pomacentridae (i.e. damselfish) in the analysis. Bites from other families (e.g. Balistidae—triggerfishes, Chaetodontidae—butterflyfishes, Labridae—wrasses) were disregarded for statistical analyses due to not contributing to herbivory functions. Data was binned into individual minutes (i.e. 1 = 00:20:00 to 00:20:59; 2 = 00:21:00 to 00:21:59; 3 = 00:22:00 to 00:22:59…) and the sum of the number of bites per functional group was calculated for each time interval (bin). A zero was recorded when no bites were recorded for a functional group in a time interval.

Generalised linear mixed effect models (GLMMs) were used to test for significant differences in bite rates between BCM-dominated and control substrates. Models were run separately for each observed group: detritivores, grazers, scrapers, all three groups combined, as well as damselfishes. Models contained bites per time interval (bin) as the response variable, treatment (factor with two levels: BCM-dominated *versus* control) as the fixed effect, and camera nested in day as random effects. The data represented count data with many zero values and initial poisson models were overdispersed. Models were accordingly specified to be zero-inflated with negative binomial distribution. This analysis was conducted in R v.3.6.1^76^ using the *glmmTMB* package^77^. Model residuals were checked using the *DHARMa* package^78^ which uses a simulation-based approach to create scaled residuals for mixed effects models which are otherwise problematic (as is the case for zero-inflated models). Plots were made using the R package *ggplot2*^79^.

### Specimen identification

Samples of the different BCM-types (morphologies) detected in the study area during benthic surveys (see “Surveys”) were collected in 50 mL scintillation vials for identification. There is still relatively little known of tropical marine benthic cyanobacteria, and most identification keys are mainly focused on freshwater species. Thus, an interdisciplinary biphasic approach combining morphology and genetics is optimal^22,68^. Accordingly, one set of samples was stored in Lugol’s iodine solution (0.4%—for microscopy), and another in ethanol (~ 96%—for sequencing). Samples were transported to the Institute for Biodiversity and Ecosystem Dynamics at the University of Amsterdam (IBED/UvA). Samples in Lugol’s iodine solution were stored in the dark at 4 °C and the ethanol samples were stored at  − 20 °C until subsequent processing.

#### Microscopy

Mat samples were extensively screened using an Olympus stereo microscope and an inverted Leica DR IMB light microscope. Phenotype determination was based on the identification keys of Komárek et al.^80^ and personal comments of Jan Kaštovský (University of South Bohemia, Czech Republic). Since we did not have the opportunity to do microscopy on fresh samples, we could only identify (reliably) to genus level, or in some cases to a higher taxonomic level. It was not possible to quantify relative dominance of different taxa from this method; this was only achievable from sequencing.

#### DNA analysis and sequencing

DNA was extracted using a PowerSoil DNA Isolation Kit (MO BIO Laboratories, Inc., Carlsbad, CA, USA) according to the protocol as described by the manufacturer. The extracted DNA was quantified using absorption spectrophotometry at 260 nm using a Nanodrop DeNovix DS11 spectrophotometer. To investigate the cyanobacterial composition of the BCM-types, the V4–V5 variable region of the 16S SSU rRNA gene (411 bp) was PCR-amplified using the universal primer set 515F: 5′-GTGYCAGCMGCCGCGGTAA -3′^81^ and 926R 5′-CCGYCAATTYMTTTRAGTTT-3′. Sequencing was performed on an Illumina MiSeq system by Molecular Research LP (Shallowater, Texas, USA), and sequences are available on NCBI SRA under Bio-project accession (PRNJA705087). Raw sequencing libraries were demultiplexed, trimmed, and tabulated using QIIME2 version 2019.10^82^. ASV tables were obtained with DADA2-denoise (parameter setting: -p-trunc-len-[fr] = 220). For taxonomic classifications using QIIME2, the reference sequences of the SILVA 16S rRNA database (99% release 132) were extracted at the appropriate primer sites, a scikit-learn naive-bayes classifier was created and the ASV sequences were classified with classify-sklearn. The 52 ASVs annotated as cyanobacteria (excluding chloroplasts) with a relative abundance of at least 1% in at least one sample were selected and blasted against the NCBI 16S rRNA database (last accessed May 2020). For phylogenetic analyses, these ASVs and the (near) full-length 16S rRNA sequences of their best-blast hits were aligned to the manually curated CyanoType database (version 1^83^), which contains 371 (near) full-length 16S rRNA nucleotide sequences. Specifically, the original CyanoType alignment was complemented with the nucleotide sequences of the best-blast hits using MAFFT (parameter settings: -add, L-INS-1 and 20PAM K = 2; version 7^84^), followed by the alignment of the 52 ASV sequences (parameter settings: -addfragment, multipair, and 20PAM K = 2). An approximate maximum-likelihood tree was obtained using FastTree using a generalised time-reversible and CAT approximation with 20 rate categories (version 2.1^85^). ITOL was used for tree visualisation (version 4^86^).

Amplicon sequencing targeting 16S provided a total of 280 ASVs within the class Cyanobacteria after application of the QIIME2 pipeline. The average sequence length was 375 bp. The average number of reads of cyanobacteria without chloroplasts was 14,110 (max 32,753, min 3843). The average total number of reads of all Archaea and Bacteria was 62,811 (max 171,750, min 37,525). The percentage of cyanobacterial reads from all reads was 22%, varying from 10 to 39%. DNA sequencing was carried out on one replicate per BCM-type.

## Supplementary Information

Supplementary Table.

Supplementary Figures.

## Data Availability

The datasets generated and analysed during the current study are available from the corresponding author on reasonable request.

## References

[1] Ford AK (2018). Reefs under siege: the rise, putative drivers, and consequences of benthic cyanobacterial mats. Front. Mar. Sci..

[2] Brocke HJ (2015). Organic matter degradation drives benthic cyanobacterial mat abundance on Caribbean coral reefs. PLoS ONE.

[3] Charpy L, Casareto BE, Langlade MJ, Suzuki Y (2012). Cyanobacteria in coral reef ecosystems: a review. J. Mar. Biol..

[4] Mangubhai S, Obura DO (2019). Silent killer: black reefs in the Phoenix Islands Protected Area. Pac. Conserv. Biol..

[5] de Bakker DM (2017). 40 years of benthic community change on the Caribbean reefs of Curaçao and Bonaire: the rise of slimy cyanobacterial mats. Coral Reefs.

[6] Albert, S., Dunbabin, M., Skinner, M., Moore, B. & Grinham, A. Benthic shift in a Solomon Islands’ lagoon: corals to cyanobacteria. In *Proceedings of the 12th International Coral Reef Symposium, Cairns, Australia, 9–13 July 2012* 1–5 (2012).

[7] Puyana M, Acosta A, Bernal-Sotelo K, Velásquez-Rodríguez T, Ramos F (2015). Spatial scale of cyanobacterial blooms in Old Providence Island Colombian Caribbean. Universitas Scientiarum.

[8] Ford AK (2017). High sedimentary oxygen consumption indicates that sewage input from small islands drives benthic community shifts on overfished reefs. Environ. Conserv..

[9] Chapra SC (2017). Climate change impacts on harmful algal blooms in US freshwaters: a screening-level assessment. Environ. Sci. Technol..

[10] Huisman J (2018). Cyanobacterial blooms. Nat. Rev. Microbiol..

[11] Gobler CJ (2020). Climate change and harmful algal blooms: insights and perspective. Harmful Algae.

[12] Wood SA (2020). Toxic benthic freshwater cyanobacterial proliferations: challenges and solutions for enhancing knowledge and improving monitoring and mitigation. Freshw. Biol..

[13] Brown KT, Bender-Champ D, Bryant DEP, Dove S, Hoegh-Guldberg O (2017). Human activities influence benthic community structure and the composition of the coral-algal interactions in the central Maldives. J. Exp. Mar. Biol. Ecol..

[14] Titlyanov EA, Yakovleva IM, Titlyanova TV (2007). Interaction between benthic algae (*Lyngbya bouillonii, Dictyota dichotoma*) and scleractinian coral *Porites lutea* in direct contact. J. Exp. Mar. Biol. Ecol..

[15] Carmichael WW (1992). Cyanobacteria secondary metabolites—the cyanotoxins. J. Appl. Bacteriol..

[16] Ritson-Williams R, Paul VJ, Bonito V (2005). Marine benthic cyanobacteria overgrow coral reef organisms. Coral Reefs.

[17] Kuffner I (2006). Inhibition of coral recruitment by macroalgae and cyanobacteria. Mar. Ecol. Prog. Ser..

[18] Kuffner IB, Paul VJ (2004). Effects of the benthic cyanobacterium *Lyngbya majuscula* on larval recruitment of the reef corals *Acropora surculosa* and *Pocillopora damicornis*. Coral Reefs.

[19] Ritson-Williams R, Arnold SN, Paul VJ (2020). The impact of macroalgae and cyanobacteria on larval survival and settlement of the scleractinian corals *Acropora palmata*, *A cervicornis* and *Pseudodiploria strigosa*. Mar. Biol..

[20] McClanahan TR (2012). Prioritizing key resilience indicators to support coral reef management in a changing climate. PLoS ONE.

[21] Cardini U, Bednarz VN, Foster RA, Wild C (2014). Benthic N_2_ fixation in coral reefs and the potential effects of human-induced environmental change. Ecol. Evol..

[22] Brocke HJ (2018). Nitrogen fixation and diversity of benthic cyanobacterial mats on coral reefs in Curaçao. Coral Reefs.

[23] Brocke HJ (2015). High dissolved organic carbon release by benthic cyanobacterial mats in a Caribbean reef ecosystem. Sci. Rep..

[24] Haas AF (2016). Global microbialization of coral reefs. Nat. Microbiol..

[25] Box SJ, Mumby PJ (2007). Effect of macroalgal competition on growth and survival of juvenile Caribbean corals. Mar. Ecol. Prog. Ser..

[26] Webster FJ, Babcock RC, Keulen MV, Loneragan NR (2015). Macroalgae inhibits larval settlement and increases recruit mortality at Ningaloo Reef, Western Australia. PLoS ONE.

[27] Barott K (2012). Natural history of coral−algae competition across a gradient of human activity in the Line Islands. Mar. Ecol. Prog. Ser..

[28] Bonaldo RM, Hay ME (2014). Seaweed-coral interactions: variance in seaweed allelopathy, coral susceptibility, and potential effects on coral resilience. PLoS ONE.

[29] Rasher DB, Hoey AS, Hay ME (2013). Consumer diversity interacts with prey defenses to drive ecosystem function. Ecology.

[30] Capper A, Cruz-Rivera E, Paul VJ, Tibbetts IR (2006). Chemical deterrence of a marine cyanobacterium against sympatric and non-sympatric consumers. Hydrobiologia.

[31] Clements KD, German DP, Piché J, Tribollet A, Choat JH (2016). Integrating ecological roles and trophic diversification on coral reefs: multiple lines of evidence identify parrotfishes as microphages. Biol. J. Linn. Soc..

[32] Cissell EC, Manning JC, McCoy SJ (2019). Consumption of benthic cyanobacterial mats on a Caribbean coral reef. Sci. Rep..

[33] Edwards CB (2014). Global assessment of the status of coral reef herbivorous fishes: evidence for fishing effects. Proc. Biol. Sci..

[34] Goatley, C., Bonaldo, R., Fox, R. & Bellwood, D. Sediments and herbivory as sensitive indicators of coral reef degradation. *Ecol. Soc.* **21**, 29 (2016).

[35] Robinson JPW (2020). Habitat and fishing control grazing potential on coral reefs. Funct. Ecol..

[36] Mouillot D (2014). Functional over-redundancy and high functional vulnerability in global fish faunas on tropical reefs. PNAS.

[37] Elmqvist T (2003). Response diversity, ecosystem change, and resilience. Front. Ecol. Environ..

[38] Duperron S (2020). New benthic cyanobacteria from Guadeloupe mangroves as producers of antimicrobials. Mar. Drugs.

[39] Bonaldo RM, Pires MM, Junior PRG, Hoey AS, Hay ME (2017). Small marine protected areas in Fiji provide refuge for reef fish assemblages, feeding groups, and corals. PLoS ONE.

[40] Ford AK (2018). Evaluation of coral reef management effectiveness using conventional versus resilience-based metrics. Ecol. Ind..

[41] Robinson JPW (2018). Environmental conditions and herbivore biomass determine coral reef benthic community composition: implications for quantitative baselines. Coral Reefs.

[42] Capper A (2016). Palatability and chemical defences of benthic cyanobacteria to a suite of herbivores. J. Exp. Mar. Biol. Ecol..

[43] Cruz-Rivera E, Paul VJ (2007). Chemical deterrence of a cyanobacterial metabolite against generalized and specialized grazers. J. Chem. Ecol..

[44] Bejarano S (2017). The shape of success in a turbulent world: wave exposure filtering of coral reef herbivory. Funct. Ecol..

[45] Lefcheck JS (2019). Tropical fish diversity enhances coral reef functioning across multiple scales. Sci. Adv..

[46] Nagle DG, Paul VJ (1998). Chemical defense of a marine cyanobacterial bloom. J. Exp. Mar. Biol. Ecol..

[47] Wilson SK, Graham NJ, Pratchett MS, Jones GP, Polunin NVC (2006). Multiple disturbances and the global degradation of coral reefs: are reef fishes at risk or resilient?. Glob. Change Biol..

[48] Pratchett MS (2006). Effects of climate-induced coral bleaching on coral-reef fishes: ecological and economic consequences. Oceanogr. Mar. Biol. Ann. Rev..

[49] Pratchett MS, Hoey AS, Wilson SK, Messmer V, Graham NAJ (2011). Changes in biodiversity and functioning of reef fish assemblages following coral bleaching and coral loss. Diversity.

[50] Potts DC (1977). Suppression of coral populations by filamentous algae within damselfish territories. J. Exp. Mar. Biol. Ecol..

[51] Mumby PJ (2012). Empirical relationships among resilience indicators on Micronesian reefs. Coral Reefs.

[52] Birrell CL, McCook LJ, Willis BL (2005). Effects of algal turfs and sediment on coral settlement. Mar. Pollut. Bull..

[53] Wismer S, Tebbett SB, Streit RP, Bellwood DR (2019). Spatial mismatch in fish and coral loss following 2016 mass coral bleaching. Sci. Total Environ..

[54] de la Morinière EC (2003). Ontogenetic dietary changes of coral reef fishes in the mangrove-seagrass-reef continuum: stable isotopes and gut-content analysis. Mar. Ecol. Prog. Ser..

[55] Komárek J (2016). A polyphasic approach for the taxonomy of cyanobacteria: principles and applications. Eur. J. Phycol..

[56] Xiao X (2014). Use of high throughput sequencing and light microscopy show contrasting results in a study of phytoplankton occurrence in a freshwater environment. PLoS ONE.

[57] Palinska KA, Surosz W (2014). Taxonomy of cyanobacteria: a contribution to consensus approach. Hydrobiologia.

[58] Li X (2020). Factors related to aggravated *Cylindrospermopsis* (cyanobacteria) bloom following sediment dredging in an eutrophic shallow lake. Environ. Sci. Ecotechnol..

[59] Taton A, Grubisic S, Brambilla E, De Wit R, Wilmotte A (2003). Cyanobacterial diversity in natural and artificial microbial mats of Lake Fryxell (McMurdo Dry Valleys, Antarctica): a morphological and molecular approach. Appl. Environ. Microbiol..

[60] Knight R (2018). Best practices for analysing microbiomes. Nat. Rev. Microbiol..

[61] Kim M, Oh H-S, Park S-C, Chun J (2014). Towards a taxonomic coherence between average nucleotide identity and 16S rRNA gene sequence similarity for species demarcation of prokaryotes. Int. J. Syst. Evol. Microbiol..

[62] Hoffmann L, Demoulin V (1993). Marine Cyanophyceae of Papua New Guinea. III. The genera Borzia and Oscillatoria. Bot. Mar..

[63] Engene N (2012). *Moorea producens* gen. nov., sp. Nov. and *Moorea bouillonii* comb. nov., tropical marine cyanobacteria rich in bioactive secondary metabolites. Int. J. Syst. Evol. Microbiol..

[64] Engene N (2013). Five chemically rich species of tropical marine cyanobacteria of the genus *Okeania* gen. nov. (Oscillatoriales, Cyanoprokaryota). J. Phycol..

[65] Komarek J, Kaštovský J, Mares J, Johansen JR (2014). Taxonomic classification of cyanoprokaryotes (cyanobacterial genera) 2014, using a polyphasic approach. Preslia.

[66] Wilmotte A, Laughinghouse HDI, Capelli C, Rippka R, Salmaso N (2017). Taxonomic Identification of Cyanobacteria by a Polyphasic Approach. Molecular Tools for the Detection and Quantification of Toxigenic Cyanobacteria.

[67] Salmaso N (2018). Diversity and cyclical seasonal transitions in the bacterial community in a large and deep perialpine lake. Microb. Ecol..

[68] Zubia M (2019). Benthic cyanobacteria on coral reefs of Moorea Island (French Polynesia): diversity response to habitat quality. Hydrobiologia.

[69] Bernard C (2017). Appendix 2: Cyanobacteria Associated with the Production of Cyanotoxins. Handbook of Cyanobacterial Monitoring and Cyanotoxin Analysis.

[70] Moritz C (2018). Status and Trends of Coral Reefs in the Pacific.

[71] Smith JE (2016). Re-evaluating the health of coral reef communities: baselines and evidence for human impacts across the central Pacific. Proc. R. Soc. B Biol. Sci..

[72] Kelly LW (2012). Black reefs: iron-induced phase shifts on coral reefs. ISME J..

[73] Bohnsack, J. A. & Bannerot, S. P. A stationary visual census technique for quantitatively assessing community structure of coral reef fishes. *NOAA Technical Report NMFS* **41**, 21 (1986).

[74] Froese, R. & Pauly, D. FishBase. World Wide Web electronic publication. www.fishbase.orghttps://www.fishbase.org/.

[75] Heenan A, Hoey AS, Williams GJ, Williams ID (2016). Natural bounds on herbivorous coral reef fishes. Proc. R. Soc. B Biol. Sci..

[76] R Development Core Team. *R: A Language and Environment for Statistical Computing.* (R Foundation for Statistical Computing, 2019).

[77] Brooks ME (2017). glmmTMB balances speed and flexibility among packages for zero-inflated generalized linear mixed modeling. R J..

[78] Hartig, F. *DHARMa: *Residual Diagnostics for Hierarchical (Multi-Level/Mixed) Regression Models. R package version 0.3.3.0. (2020). http://florianhartig.github.io/DHARMa/

[79] Wickham H (2016). ggplot2: Elegant Graphics for Data Analysis.

[80] Komárek J, Anagnostidis K (2005). Cyanoprokaryota 2.Teil: Oscillatoriales.

[81] Quince C, Lanzen A, Davenport RJ, Turnbaugh PJ (2011). Removing noise from pyrosequenced amplicons. BMC Bioinform..

[82] Bolyen E (2019). Reproducible, interactive, scalable and extensible microbiome data science using QIIME 2. Nat. Biotechnol..

[83] Ramos V, Morais J, Vasconcelos VM (2017). A curated database of cyanobacterial strains relevant for modern taxonomy and phylogenetic studies. Sci. Data.

[84] Katoh K, Rozewicki J, Yamada KD (2019). MAFFT online service: multiple sequence alignment, interactive sequence choice and visualization. Brief. Bioinform..

[85] Price MN, Dehal PS, Arkin AP (2010). FastTree 2: approximately maximum-likelihood trees for large alignments. PLoS ONE.

[86] Letunic I, Bork P (2019). Interactive tree of life (iTOL) v4: recent updates and new developments. Nucl. Acids Res..

