# Supplementary material for: First insights into the impacts of benthic cyanobacterial mats on fish herbivory functions on a nearshore coral reef
Source: Sci Rep. 2021 Mar 30;11:7147. doi: 10.1038/s41598-021-84016-z (PMC8009962; doi:10.1038/s41598-021-84016-z)
Supplement: Supplementary file 1 — Supplementary Table. [file 41598_2021_84016_MOESM1_ESM.pdf]

First insights into the impacts of benthic cyanobacterial mats on fish herbivory functions on a nearshore coral reef

Amanda K Ford[1,2], Petra M Visser[3], Maria J van Herk[3], Evelien Jongepier[4], Victor Bonito[5]

Supplementary Table

Table S1. List of observed species and corresponding functional groups (based on those described by Heenan et al. 2016*).

**Species**	**Functional group**
Kyphosus vaigiensis	Browser
Naso lituratus	Browser
Naso unicornis	Browser
Ctenochaetus binotatus	Detritivore
Ctenochaetus striatus	Detritivore
Acanthurus blochii	Grazer
Acanthurus lineatus	Grazer
Acanthurus nigricauda	Grazer
Acanthurus sp.	Grazer
Acanthurus triostegus	Grazer
Siganus punctatus	Grazer
Siganus spinus	Grazer
Zebrasoma scopas	Grazer
Zebrasoma velifer	Grazer
Chlorurus bleekeri	Scraper
Chlorurus sp.	Scraper
Scarus altipinnis	Scraper
Scarus dimidiatus	Scraper
Scarus oviceps	Scraper
Scarus psitticus	Scraper
Scarus rivulatus	Scraper
Scarus schlegeli	Scraper
Scarus sp.	Scraper

**Heenan, A., Hoey, A. S., Williams, G. J. & Williams, I. D. Natural bounds on herbivorous coral reef fishes. Proc. R. Soc. B Biol. Sci. 283, 20161716 (2016).*
