# Supplementary material for: First insights into the impacts of benthic cyanobacterial mats on fish herbivory functions on a nearshore coral reef
Source: Sci Rep. 2021 Mar 30;11:7147. doi: 10.1038/s41598-021-84016-z (PMC8009962; doi:10.1038/s41598-021-84016-z)
Supplement: Supplementary file 2 — Supplementary Figures. [file 41598_2021_84016_MOESM2_ESM.pdf]

First insights into the impacts of benthic cyanobacterial mats on fish herbivory functions on a nearshore coral reef

Amanda K Ford[1,2], Petra M Visser[3], Maria J van Herk[3], Evelien Jongepier[4], Victor Bonito[5]

Supplementary Figures

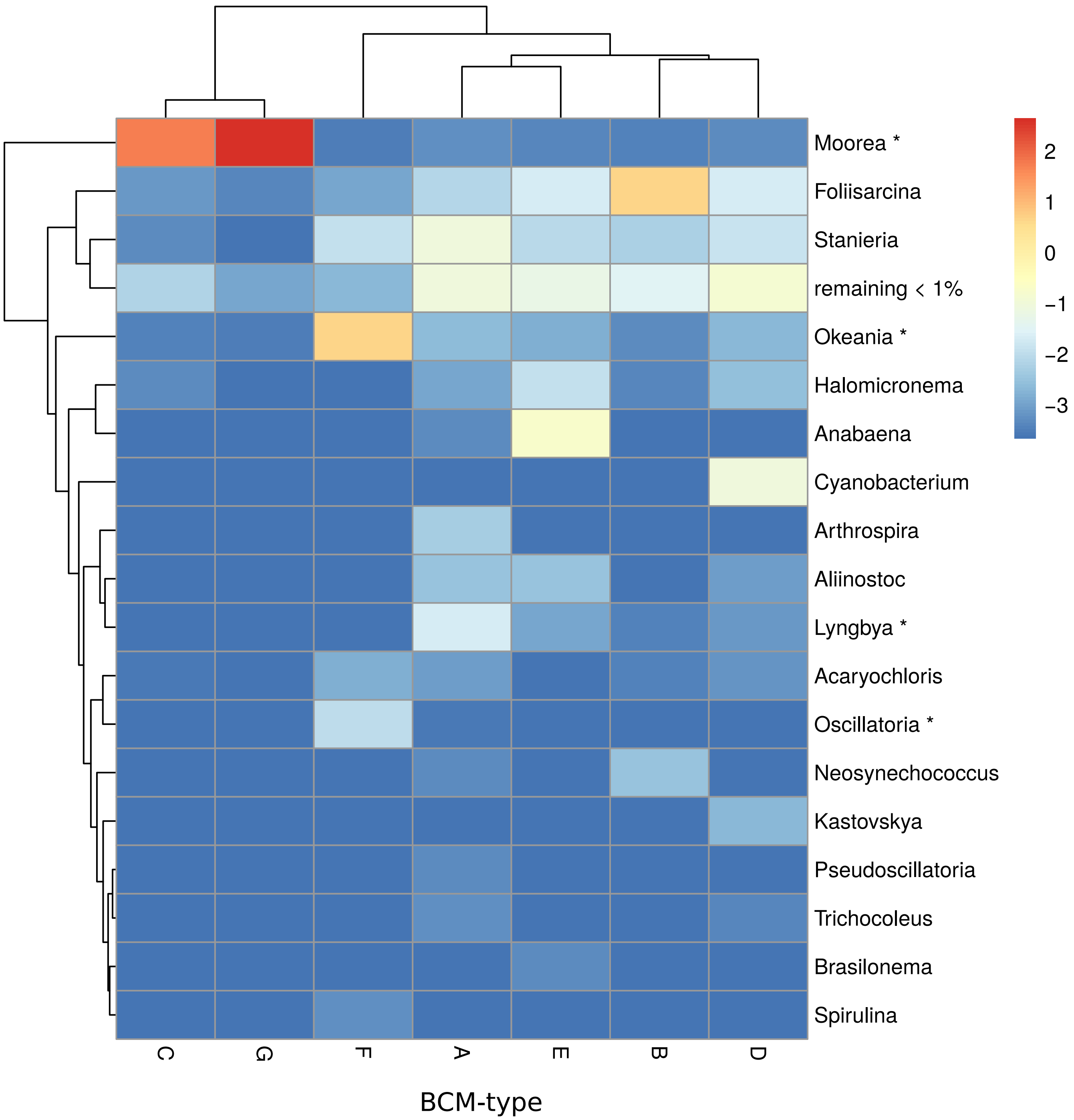

Figure S1. Heatmap of logit-transformed relative abundance of cyanobacterial genera. Quantified by 16S rRNA amplicon sequencing and annotated using BLAST. Genera with >95% best-blast similarity are marked with *. Only features with a relative abundance of >1% in at least one sample were included. Dendrograms are based on Euclidean distances of normalized relative abundances.

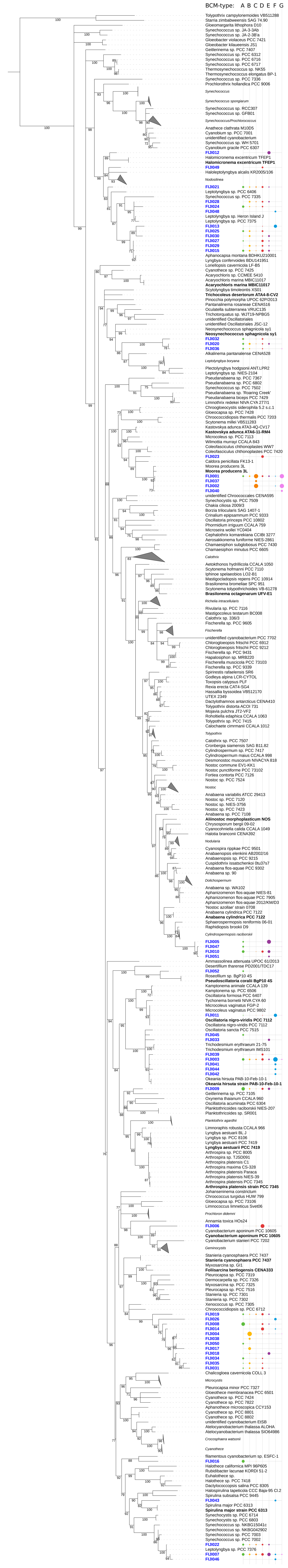

Figure S2. Approximate maximum-likelihood phylogeny of the most abundant features found in the Fiji cyanobacterial mats. Features with a relative abundance of > 1% in at least one sample are included (blue labels). Reference set (black labels) alignments come from manually curated CyanoType database (http://lege.ciimar.up.pt/cyanotype/), complemented with best-blast hits (bold font). Circle sizes are proportional to the relative abundance of each feature in each of the seven identified BCM-types.
